# Mangrove floristic composition dataset of the Setiu Lagoon, Terengganu Malaysia

**DOI:** 10.1016/j.dib.2022.108020

**Published:** 2022-03-04

**Authors:** Siti Mariam Muhammad Nor, Mohamad Saiful Imran Sahari, Nadiatul Azimah Mohd Razali, Muhamad Razali Salam, Wan Fatihah Wan Mustaffa, Elton Roy Stephen, Rohmansyah Mohd Yusof, Puteri Nurnajmi Jamaludin, Puteri Nurnaim Jamaludin, Norlida Mokhetar

**Affiliations:** a Faculty of Science and Marine Environment, Universiti Malaysia Terengganu, Kuala Nerus, Terengganu 21030, Malaysia; b Mangrove Research Unit (MARU), Institute of Oceanography and Environment, Universiti Malaysia Terengganu, Kuala Nerus, Terengganu 21030, Malaysia

**Keywords:** Mangroves flora, Lagoon, Mix mangrove species, East coast of Peninsular Malaysia

## Abstract

This article documents the composition of plant species collected from the mangrove forest of the Setiu Lagoon, Terengganu. The data was collated between 2004 and 2019 through fieldwork to compile an inventory of mangrove flora on 13 islands located in the Setiu Lagoon. Field inventories using circular sample plots of 5.6 m radius and square plots of 100 m × 20 m were employed in the study. In each plot, the mangrove plant species were enumerated and identified. A total of 74 species belonging to 39 families were recorded, comprising of 23 exclusive, 49 non-exclusive and 2 associate mangrove species. Pulau Telaga Tujuh supported the most diverse mangrove with 47 species in contrast to Pulau Tok Ba and Pulau Gemia that supported the least number of mangroves with only 10 species recorded each. The Lagoon is also a home to critically endangered species, *Olax psittacorum* (Lam) Vahl and an interesting species, *Hydnophytum formicarum* Jack (an ant plant, the mangroves epiphyte). Overall, islands of the Setiu Lagoon supported a mix of mangrove plant species which were exclusive and non-exclusive. The present work provides important baseline data for management, conservation and restoration of mangrove forest in the east coast of Peninsular Malaysia. Various mangrove plant species including the presence of hybrid species underlines the importance of the Setiu Lagoon as a center of mangrove plant diversity, particularly in the Southeast Asia region.

**Specifications Table**

**Table:** 

Subject	Biodiversity
Specific subject area	Mangrove plant diversity
Type of data	Table
How data were acquired	Mangrove inventory study, circular plots of 5.6 m radius and square plots of 100 m × 20 m. Mangrove habitat species enumeration and identification. Data obtained from circular plots were derived from the mangrove inventory work whilst the data collected using square plots were used to study the mangrove ecology.
Data format	Raw
Description of data collection	The data were obtained through fieldworks to document an inventory of mangrove plant species on 13 islands of the Setiu Lagoon, Terengganu between 2004 and 2019. The data were furthered classified based on their taxonomy group (family, genus and species) including mangrove exclusivity and conservation status.
Data source location	Setiu Lagoon, Terengganu, East Coast of Peninsular Malaysia. Pulau Telaga Tujuh: 5°41′42.0"N 102°41′51.2"E Pulau Tok Haji: 5°39′31.3"N 102°44′14.8"E Pulau Busung: 5°39′55.4"N 102°44′01.6"E Pulau Semut: 5°41′16.1"N 102°42′18.1"E Pulau Layat: 5°41′49.6"N 102°41′41.3"E Pulau Che Hamid: 5°40′25.7"N 102°43′15.9"E Pulau Besar: 5°40′14.1"N 102°43′29.6"E Pulau Rhu: 5°38′30.2"N 102°46′08.1"E Pulau Tok Ba: 5°37′41.0"N 102°47′00.5"E Pulau Ubi: 5°37′59.8"N 102°46′28.5"E Pulau Sutung: 5°39′02.3"N 102°45′16.9"E Pulau Gemia: 5°38′57.2"N 102°45′05.7"E Pulau Che Him: 5°40′56.3"N 102°42′34.1"E
Data accessibility	With the article

**Value of the Data**

• This is a mangrove floristic dataset covering 13 small islands of Setiu Lagoon, Terengganu, Malaysia.
• The checklist allows researchers to appraise the mangrove floristic composition in a Malaysian mangrove-lagoon setting.
• The checklist is also important as a database for mangrove habitat biodiversity resources in east coast of Peninsular Malaysia.
• Conservation status of mangrove vegetations may reveal the importance of mangrove forest in the Setiu lagoon as among the unique elements of Setiu Wetland State Park for mangrove conservation and protection.
• The dataset may also serve as baseline documentation for mangrove habitat species composition which can be used in future research, particularly in countering forest degradation.
• The mangrove floristic list is crucial for carbon stock estimation while emphasizing the mangrove ecosystem's significant role in mitigating climate change and global warming.

## 1 Data

The dataset presents the mangrove species from 13 out of 17 small islands of the Setiu Lagoon, Terengganu. This is the first study documenting mangrove species in a lagoon mangrove forest setting in Malaysia. The checklist is presented in Table 1. Additional information such as that on mangrove habitat exclusivity (classification of mangrove species originality) is available in [1], while their conservation status is referred to in [2], and in Table 2. A summary of the dataset appears in Table 3.

**Table 1:** List of mangrove floristic species on 13 small islands in the Setiu Lagoon, Terengganu, Malaysia.

Family	Scientific Name	Exclusivity	PL	PTT	PCH	PBe	PBu	PTH	PR	PTB	PU	PSu	PG	PSe	PCh
Acanthaceae	*Avicennia alba* Blume	Exclusive	2011	2011	2004	2004	2004; 2019	2004; 2019	-	-	2004	2004	2004	2011; 2019	2010
Acanthaceae	*Avicennia rumphiana* Hallier f.	Exclusive	2011	2011	-	-	-	-	2004	2004	-	-	-	2011	-
Acanthaceae	*Acanthus ilicifolius* Lour	Exclusive	-	-	2004	-	-	-	-	-	-	-	-	-	-
Aizoceae	*Sesuvium portulacastrum* (L.) L.	Non-Exclusive	-	-	2004	-	-	-	-	-	-	-	-	-	-
Apocynaceae	*Finlaysonia obovata* Wall.	Exclusive	2011	-	-	-	-	-	-	-	-	-	-	2011	-
Apocynaceae	*Cerbera manghas* L.	Non-Exclusive	-	2011	-	-	-	-	-	-	-	-	-	-	-
Apocynaceae	*Cerbera odollam* Gaertn.	Non-Exclusive	-	2011	-	-	-	-	-	-	-	-	-	-	-
Apocynaceae	*Dischidia nummularia* R. Br.	Non-Exclusive	-	2011	-	-	-	-	-	-	-	-	-	-	-
Apocynaceae	*Secamone elliptica* R. Br.	Non-Exclusive	2011	-	-	-	-	-	-	-	-	-	-	-	-
Arecaceae	*Nypa fruticans* Wurmb	Exclusive	2011	2011	-	2004	2004; 2019	2004; 2019	2004	2004	2004	2004	2004	2011	2010
Arecaceae	*Calamus erinaceus* (Becc.) J. Dransf.	Non-Exclusive	-	-	-	-	-	2019	-	-	-	-	-	-	-
Arecaceae	*Cocos nucifera* L.	Non-Exclusive	-	2011	-	-	-	-	-	-	-	-	-	-	2010
Aspleniaceae	*Asplenium nidus* L.	Associate	2011	-	-	-	-	-	-	-	-	-	-	-	-
Asteracaeae	*Wollastonia biflora* (L) DC	Non-Exclusive	-	2011	-	-	-	-	-	-	-	-	-	-	-
Bignoniaceae	*Dolichandrone spathacea* (L.f.) Seem*.*	Non-Exclusive	-	-	-	-	-	-	-	-	2004	2004	-	-	-
Calophyllaceae	*Calophyllum rupicola* Ridl.	Non-Exclusive	-	-	-	-	-	2004	-	-	-	-	-	-	-
Casuarinaceae	*Casuarina equisetifolia* L. J.R. Forst. & G. Forst.	Non-Exclusive	-	2011	2004	-	-	2004	-	-	-	-	-	-	2010
Celastraceae	*Salacia chinensis* L.	Non-Exclusive	2011	2011	-	-	-	-	-	-	-	-	-	-	-
Clusiaceae	*Garcinia hombroniana* Pierre	Non-Exclusive	-	-	-	-	-	2004	-	-	-	-	-	-	-
Combretaceae	*Terminalia catappa* L.	Non-Exclusive	2011	2011	-	-	-	-	2004	-	-	-	-	-	2010

Combretaceae	*Lumnitzera racemosa* Willd.	Exclusive	-	-	2004	-	-	-	-	-	2004	-	-	-	-
Combretaceae	*Lumnitzera littorea* (Jack) Voight	Exclusive	-	-	-	2004	-	-	-	-	-	-	-	2019	-
Convolvulaceae	*Ipomoea pes-caprae* (L) R. Br.	Non-Exclusive	-	2011	-	-	-	-	-	-	-	-	-	-	-
Cyperaceae	*Fimbristylis ferruginea* (L.) Vahl.	Associate	-	-	2004	-	-	-	-	-	-	-	-	-	-
Dipterocarpaceae	*Vatica pauciflora* (Korth.) Blume	Non-Exclusive	2011	-	-	-	-	-	-	-	-	-	-	-	-
Ebenaceae	*Diospyros ferrea* (Willd) Bakh.	Non-Exlusive	-	2011	-	-	-	-	-	-	-	-	-	-	-
Euphorbiaceae	*Excoecaria agallocha* L.	Exclusive	2011	2011	2004	2004	2004; 2019	2004; 2019	-	2004	2004	2004	2004	2011; 2019	2010
Euphorbiaceae	*Glochidion littorale* Blume	Non-Exclusive	-	-	-	-	-	-	-	-	2004	-	-	-	-
Fabaceae	*Cynometra iripa* Kostel	Non-Exclusive	-	2011	-	-	-	-	-	-	-	-	-	-	-
Fabaceae	*Dalbergia candenatensis* (Dennst) Prain	Non-Exclusive	-	2011	-	-	-	-	-	-	-	-	-	-	-
Fabaceae	*Caesalpinia crista* L.	Non-Exclusive	2011	-	-	-	-	-	-	-	-	-	-	-	-
Fabaceae	*Pongamia pinnata* (L.) Pierre	Non-Exclusive	-	2011	-	-	-	-	-	2004	-	-	-	-	-
Fabaceae	*Derris trifoliata* Lour.	Non-Exclusive	2011	2011	-	-	-	-	-	-	-	-	-	2011	-
Fabaceae	*Aganope heptaphylla* (L.) Polhill.	Non-Exclusive	2011	-	-	-	-	-	-	-	-	2004	-	-	-
Fabaceae	*Dendrolobium umbellatum* (L.) Benth*.*	Non-Exclusive	2011	2011	-	-	-	-	-	-	-	-	-	2011; 2019	-
Fabaceae	*Intsia bijuga (Colebr.)* Kuntze	Exclusive	2011	2011	-	-	-	-	-	-	-	-	-	2011	-
Flagellariaceae	*Flagellaria indica* L*.*	Non-Exclusive	2011	2011	-	-	-	-	-	-	-	-	-	-	-
Goodeniaceae	*Scaevola taccada* (Gaertn.) Roxb	Non-Exclusive	-	2011	2004	-	-	-	-	-	-	-	-	-	-
Lamiacaea	*Volkameria inermis* L.	Non-Exclusive	2011	2011	2004	-	-	-	-	-	2004	-	-	2011; 2019	-
Lamiaceae	*Premna serratifolia* L.	Non-Exclusive	-	2011	-	-	-	-	-	-	-	2004	-	-	-
Lauraceae	*Neolitsea zeylanica* (Nees & T.Nees) Merr.	Non-Exclusive	-	2011	-	-	-	-	-	-	-	-	-	-	-

Lecythidaceae	*Barringtonia racemosa* (L) Spreng.	Non-Exclusive	-	2011	-	-	-	-	-	-	-	2004	-	-	-
Lythraceae	*Sonneratia alba* J.J.Sm. Griff.	Exclusive	2011	-	-	-	-	-	2004	2004	2004	-	-	2011	-
Lythraceae	*Sonneratia caseolaris* (L.) Engler	Exclusive	-	-	-	-	-	-	-	-	-	-	-	2011	-
Malvaceae	*Talipariti tiliaceum* (L.) Fryxell.	Non-Exclusive	2011	2011	-	2004	-	2004; 2019	2004	-	2004	2004	2004	2011; 2019	2010
Malvaceae	*Thespesia populnea* (L.) Soland. Ex Correa	Non-Exclusive	2011	2011	-	-	-	-	-	-	-	-	-	-	-
Malvaceae	*Heritiera littoralis* Aiton	Exclusive	2011	2011	-	2004	2004; 2019	2004; 2019	2004	2004	-	2004	2004	2019	2010
Melastomataceae	*Memecylon edule* Roxb.	Non-Exclusive	-	2011	-	-	-	-	-	-	-	2004	-	-	-
Meliaceae	*Xylocarpus granatum* (L.) Koenig	Exclusive	2011	2011	-	2004	2004; 2019	2004; 2019	-	2004	-	2004	-	2011; 2019	2010
Myrtaceae	*Syzygium kunstleri* (King) Bahadur & R.C. Gaur	Non-Exclusive	-	-	-	-	-	-	-	-	-	2004	2004	-	-
Olacaceae	*Olax psittacorum* (Lam) Vahl.	Non-Exclusive	-	2011	-	-	-	-	-	-	-	-	-	-	-
Orchidaceae	*Dendrobium crumenatum* Sw.	Non-Exclusive	-	2011	-	-	-	-	-	-	-	-	-	-	-
Orchidaceae	*Taeniophyllum pusillum* (Willd.) Seidenf. & Ormerod	Non-Exclusive	-	2011	-	-	-	-	-	-	-	-	-	2011	-
Pandanaceae	*Pandanus tectorius* Parkinson	Non-Exclusive	2011	2011	2004	2004	-	2004; 2019	-	-	-	2004	-	2011; 2019	-
Poaceae	*Spinifex littoreus* (Burm.f.) Merr.	Non-Exclusive	-	2011	-	-	-	-	-	-	-	-	-	-	-
Poaceae	*Zoysia matrella* (L.) Merr*.*	Non-Exclusive	-	-	2004	2004	-	-	-	-	-	-	-	-	-
Polypodiaceae	*Pyrrosia piloselloides* (L.) M.G. Price	Non-Exclusive	2011	-	-	-	-	-	-	-	-	-	-	-	-
Primulaceae	*Aegiceras corniculatum* (L.) Blanco	Exclusive	-	-	-	-	2019	-	-	-	-	-	-	2011; 2019	-
Rhizophoraceae	*Carallia brachiata* (Lour.) Merr.	Non-Exclusive	-	-	-	-	-	2004	-	-	-	-	-	-	-
Rhizophoraceae	*Bruguiera gymnorhiza* (L.) Lam. Ex Savigny	Exclusive	2011	2011	2004	2004	2004; 2019	2004; 2019	2004	2004	2004	-	-	2011; 2019	2010

Rhizophoraceae	*Bruguiera cylindrica* (L.) Blume	Exclusive	2011	2011	2004	2004	2004; 2019	2004; 2019	2004	-	2004	2004	2004	2011; 2019	2010
Rhizophoraceae	*Bruguiera hainesii* C.G. Rogers	Exclusive	2011	-	-	-	-	-	-	-	-	-	-	-	-
Rhizophoraceae	*Ceriops zippeliana* Blume	Exclusive	2011	2011	2004	2004	2004; 2019	2004; 2019	2004	2004	2004	2004	2004	2011; 2019	2010
Rhizophoraceae	*Ceriops tagal* (Perr.) C.B.Rob.	Exclusive	2011	2011	-	-	2019	-	-	-	-	-	-	2011; 2019	2010
Rhizophoraceae	*Rhizophora apiculata* Blume	Exclusive	2011	2011	2004	2004	2004; 2019	2004; 2019	2004	2004	2004	2004	2004	2011; 2019	2010
Rhizophoraceae	*Rhizophora x annamalayana*	Exclusive	2011	-	-	-	-	-	-	-	-	-	-	-	-
Rhizophoraceae	*Rhizophora mucronata* Lam.	Exclusive	-	-	-	-	-	-	-	-	-	-	-	2011	-
Rhizophoraceae	*Brugueira sexangula* (Lour.) Poir	Exclusive	-	-	-	-	-	-	2004	-	2004	2004	2004	2011; 2019	-
Rubiaceae	*Hydnophytum formicarum* Jack	Non-Exclusive	-	2011	-	-	-	-	-	-	-	-	-	-	-
Rubiaceae	*Morinda citrifolia* L.	Non-Exclusive	-	2011	-	-	-	-	-	-	-	-	-	-	-
Rubiaceae	*Myrmecodia tuberosa* Jack	Non-Exclusive	-	2011	-	-	-	-	-	-	-	-	-	-	-
Sapindaceae	*Allophylus cobbe* (L.) Raeusch	Non-Exclusive	2011	2011	-	-	-	-	-	-	-	2004	-	-	-
Sapotaceae	*Planchonella obovata* (R.Br.) Pierre	Non-Exclusive	2011	2011	-	-	-	2004	-	-	-	2004	-	-	-
Ximeniaceae	*Ximenia americana* L.	Non-Exclusive	-	2011	-	-	-	-	-	-	-	-	-	-	-

			33	47	15	13	11	17	11	10	14	19	10	25	14

The distribution of each species within the Setiu Lagoon was recorded on the islands abbreviated as PL=Pulau Layat, PTT= Pulau Telaga Tujuh, PCH=Pulau Che Hamid, PBe= Pulau Besar, PBu=Pulau Busung, PTH= Pulau Tok Haji, PR=Pulau Rhu, PTB=Pulau Tok Ba, Pu=Pulau Ubi, PSu=Pulau Sutung, PG= Pulau Gemia, PSe=Pulau Semut, PCh=Pulau Che Him.

**Table 2:** Conservation status of mangrove habitat species in the Setiu Lagoon, Terengganu, Malaysia.

Family/Species	Common Name	IUCN Status	IUCN Year of Assessment
**ACANTHACEAE**			
*Avicennia alba* Blume	Api-api/Api-api hitam	Least Concern	2008
*Avicennia rumphiana* Hallier f.	Api-api bulu	Vulnerable	2008
*Acanthus ilicifolius* Lour	Jeruju hitam/ Jeruju Putih	Least Concern	2010
**AIZOCEAE**			
*Sesuvium portulacastrum* (L.) L	Gelang laut/Gelang Pasir	Least Concern	2020
**APOCYNACEAE**			
*Finlaysonia obovata* Wall.	Kalak Kambing/Pelir kambing	Not Evaluated	-
*Cerbera manghas* L.	Pong-pong	Least Concern	2018
*Cerbera odollam* Gaertn.	Pong-pong/Kepompong	Least Concern	2018
*Dischidia nummularia* R.Br.	Duit-duit	Not Evaluated	-
*Secamone elliptica* R. Br.	-	Not Evaluated	-
**ARECACEAE**			
*Nypa fruticans* Wurmb.	Nipah	Least Concern	2008
*Calamus erinaceus* (Becc.) J. Dransf.	Rotan	Not Evaluated	-
*Cocos nucifera* L.	Kelapa	Not Evaluated	-
**ASPLENIACEAE**			
*Asplenium nidus* L.	Paku langsuyar/Daun Semun	Not Evaluated	-
**ASTERACAEAE**			
*Wollastonia biflora* (L) DC	Serunai laut/Pokok Serunai	Not Evaluated	-
**BIGNONIACEAE**			
*Dolichandrone spathacea* (L.f.) Seem.	Tui/Taring Buaya	Least Concern	2008
**CALOPHYLLACEAE**			
*Calophyllum rupicola* Ridl.	Bintangor laut	Not Evaluated	-
**CASUARINACEAE**			
*Casuarina equisetifolia* L. J.R. Forst. & G. Forst.	Rhu laut/Rhu Pantai	Least Concern	2018
**CELASTRACEAE**			
*Salacia chinensis* L.	Rakaat Kecil	Not Evaluated	-
**CLUSIACEAE**			
*Garcinia hombroniana* Pierre	Beruas/Manggis Hutan	Not Evaluated	-
**COMBRETACEAE**			
*Terminalia catappa* L.	Ketapang/Jelawai Ketapang	Least Concern	2018
*Lumnitzera racemosa* Willd.	Teruntum bunga putih	Least Concern	2008
*Lumnitzera littorea* (Jack) Voight	Teruntum merah	Least Concern	2008
**CONVOLVULACEAE**			
*Ipomoea pes-caprae* (L) R. Br.	Tapak kuda	Least Concern	2020
**CYPERACEAE**			
*Fimbristylis ferruginea* (L.) Vahl.	Rumput	Least Concern	2019
**DIPTEROCARPACEAE**			
*Vatica pauciflora* (Korth.) Blume	Resak paya/Resak Laru	Vulnerable	2017
**EBENACEAE**			
*Diospyros ferrea* (Willd) Bakh.	Kayu Arang	Not Evaluated	-
**EUPHORBIACEAE**			
*Excoecaria agallocha* L.	Buta-buta	Least Concern	2008
*Glochidion littorale* Blume	Jambu kera/Selunsur	Least Concern	2018
**FABACEAE**			
*Cynometra iripa* Kostel	Kekatong laut	Least Concern	2008
*Dalbergia candenatensis* (Dennst) Prain	Api-api jambu	Not Evaluated	-
*Caesalpinia crista* L.	Kuku tupai/Mata Kijang	Not Evaluated	-
*Pongamia pinnata* (L.) Pierre	Mempari	Least Concern	2010
*Derris trifoliata* Lour.	Ketui	Not Evaluated	-
*Aganope heptaphylla* (L.) Polhill.	Ketui besar, Omis-Omis	Not Evaluated	-
*Dendrolobium umbellatum* (L.) Benth.	Petai laut	Least Concern	2020
*Intsia bijuga* (Colebr.) Kuntze	Merbau ipil	Near Threatened	2020
**FLAGELLARIACEAE**			
*Flagellaria indica* L.	Rotan Tikus/Rotan Kera	Not Evaluated	-
**GOODENIACEAE**			
*Scaevola taccada* (Gaertn.) Roxb	Akar Pahit/Buas-Buas Laut	Not Evaluated	-
**LAMIACEAE**			
*Volkameria inermis* L.	Lampin budak	Not Evaluated	-
*Premna serratifolia L.*	Buas-buas/Bangkung Kayu	Least Concern	2020
**LAURACEAE**			
*Neolitsea zeylanica* (Nees & T.Nees) Merr.	Teja pasir/Medang Pasir	Least Concern	2020
**LECYTHIDACEAE**			
*Barringtonia racemosa (*L) Spreng.	Putat ayam/Putat Kedul	Least Concern	2019
**LYTHRACEAE**			
*Sonneratia alba* J.J.Sm. Griff.	Perepat	Least Concern	2008
*Sonneratia caseolaris* (L.) Engler	Berembang	Least Concern	2008
**MALVACEAE**			
*Talipariti tiliaceum* (L.) Fryxell.	Bebaru/ Baru Laut	Least Concern	2017
*Thespesia populnea* (L.) Soland. Ex Correa	Bebaru	Least Concern	2017
*Heritiera littoralis* Aiton	Dungun	Least Concern	2008
**MELASTOMATACEAE**			
*Memecylon edule* Roxb.	Nipis Kulit/Delek Putih	Not Evaluated	-
**MELIACEAE**			
*Xylocarpus granatum* (L.) Koenig	Nyireh bunga	Least Concern	2008
**MYRTACEAE**			
*Syzygium kunstleri* (King) Bahadur & R.C.Gaur	Kelat sp.	Not Evaluated	-
**OLACACEAE**			
*Olax psittacorum* (Lam) *Vahl.*	Kodak acing	Critically Endangered	1998
**ORCHIDACEAE**			
*Dendrobium crumenatum* Sw.	Anggerik/ Pokok merpati	Not Evaluated	-
*Taeniophyllum pusillum* (Willd.) Seidenf. & Ormerod	Orkid hantu	Not Evaluated	-
**PANDANACEAE**			
*Pandanus tectorius* Parkinson	Mengkuang Duri/Pandan laut	Least Concern	2018
**POACEAE**			
*Spinifex littoreus* (Burm.f.) Merr.	Rumput Bebaling	Not Evaluated	-
*Zoysia matrella* (L.) Merr.	Rumput manila	Not Evaluated	-
**POLYPODIACEAE**			
*Pyrrosia piloselloides* (L.) M.G.Price	Sisik naga/ Sakat Ribu-Ribu	Not Evaluated	-
**PRIMULACEAE**			
*Aegiceras corniculatum* (L.) Blanco	Kuku helang/ Kacang-Kacang	Least Concern	2008
**RHIZOPHORACEAE**			
*Carallia brachiate* (Lour.) Merr.	Meransi/Merpuing	Not Evaluated	-
*Bruguiera gymnorhiza* (L.) Lam. Ex Savigny	Tumu merah/Berus Merah	Least Concern	2008
*Bruguiera cylindrica* (L.) Blume	Bakau putih/Berus Putih	Least Concern	2008
*Ceriops zippeliana* Blume	Tengar	Least Concern	2008
*Ceriops tagal* (Perr.) C.B.Rob.	Tengar Samak	Least Concern	2008
*Rhizophora apiculata* Blume	Bakau minyak	Least Concern	2008
*Rhizophora* x *annamalayana*	Bakau kacuk	Not Evaluated	-
*Rhizophora mucronata* Lam.	Bakau kurap	Least Concern	2008
*Bruguiera sexangula* (Lour.) Poir	Tumu Putih/Tumu Berau	Least Concern	2008
**RUBIACEAE**			
*Hydnophytum formicarum* Jack	Sarang semut/Kepala Beruk	Not Evaluated	-
*Morinda citrifolia* L.	Mengkudu/Kemedu	Not Evaluated	-
*Myrmecodia tuberosa* Jack	Periuk hantu	Not Evaluated	-
**SAPINDACEAE**			
*Allophylus cobbe* (L.) Raeusch	Kasai Daun Kecil	Not Evaluated	-
**SAPOTACEAE**			
*Planchonella obovata* (R.Br.) Pierre	Nyatoh Laut	Not Evaluated	-
**XIMENIACEAE**			
*Ximenia americana* L.	Bedara Laut	Least Concern	2018

**Table 3:** Summary of the dataset.

		Exclusivity			Conservation Status				
Family	Species	Exclusive	Non-Exclusive	Associate	CE	VU	NT	LC	NE
39	74	23	49	2	1	2	1	37	32

Conservation status is abbreviated as follows: CE=critically endangered, VU=vulnerable, NT=near threatened, LC=Least Concern, NE=not evaluated.

## 2 Experimental Design, Materials and Method

The inventory data were obtained from fieldworks in the mangrove forest using circular and square sampling plots. Circular plots were employed for the tree inventory in the mangrove forest [3] whilst square plots were used for the study of biomass and carbon stock. Plants were numbered in each plot and identifications were made *in situ* whenever possible. However, samples were also collected and brought back to the laboratory for further identification against various references [4], [5], [6], including a comparison to herbarium specimens. Identification was made to the level of family, genus and species, with the most recent names used in their classifications adopted [7,8].

## CRediT authorship contribution statement

**Siti Mariam Muhammad Nor:** Writing – original draft, Investigation, Data curation. **Mohamad Saiful Imran Sahari:** Data curation, Formal analysis. **Nadiatul Azimah Mohd Razali:** Data curation, Formal analysis. **Muhamad Razali Salam:** Data curation, Methodology. **Wan Fatihah Wan Mustaffa:** Data curation. **Elton Roy Stephen:** Data curation. **Rohmansyah Mohd Yusof:** Data curation. **Puteri Nurnajmi Jamaludin:** Data curation. **Puteri Nurnaim Jamaludin:** Data curation. **Norlida Mokhetar:** Data curation.

## Declaration of Competing Interest

The authors declare that they have no known competing financial interests or personal relationships that could have appeared to influence the work reported in this paper.

## Appendix Supplementary materials

Image, application 1

## References

[1] Japar S.B. (1994). Proceedings of the Third Asean-Australia Symposium on Living Coastal Resources.

[2] IUCN, (2021). https://www.iucnredlist.org/.

[3] Shamsudin I., Ismail H, Nasir H. (1999). Proceedings of the Seminar Penerangan dan Pengurusan Hutan Paya Laut Negeri Johor, Johor Bharu.

[4] M.H. Lokman, I. Sulong, Mangroves of Terengganu, Kolej Universiti Sains and Teknologi Malaysia & Forestry Department Peninsular Malaysia, Terengganu, 2001, pp. 135.

[5] MYBIS 2021. Malaysia Biodiversity Information System (MYBIS), http://www.mybis.gov.my. Accessed August 19, 2021.

[6] Wan J.W.A., Norhayati A., Abdul-Latiff M. (2018).

[7] POWO 2021. Plants of the World Online. Facilitated by Royal Botanic Gardens, Kew. Published on the Internet; http://www.plantsoftheworldonline.org/. Accessed May 24, 2021.

[8] IPNI 2021. The International Plant Names Index. http://www.ipni.org. Accessed March 20, 2021.

